# Assessment of Formate Dehydrogenase Stress Stability *In vivo* using Inactivation by Hydrogen Peroxide

**Published:** 2010-04

**Authors:** S.S. Savin, V.I. Tishkov

**Affiliations:** Bach Institute of Biochemistry, Russian Academy of Sciences; Innovations and High Technologies MSU Ltd.; Division of Chemical Enzymology, Department of Chemistry, Lomonosov Moscow State University

**Keywords:** formate dehydrogenase, hydrogen peroxide, inactivation, stress, mutant enzyme

## Abstract

Abstract Kinetic studies on hydrogen peroxide–induced inactivation of mutant formate dehydrogenase from *Pseudomonas sp.* 101 (PseFDH Cys255Ala) suggest a simple bimolecular mechanism for enzyme reaction with the inactivation agent. In the excess of hydrogen peroxide, the decrease in enzyme activity follows first–order kinetics. Therefore, the first–order effective inactivation kinetic constants determined for various FDH forms at a constant H_2_O_2_ concentration can be used as a quantitative measure of the enzyme stability. It was shown that two cysteine residues located in the active site formate– and coenzyme–binding domains (Cys145 and Cys255, respectively) make similar contributions to the enzyme stability, while the contribution of Cys354 is insignificant. The inactivation kinetics of wild–type PseFDH, mutant PseFDH Cys145Ser/Cys255Ala, and FDH produced under stress conditions by bacterium *Staphylococcus aureus*, higher plants *Arabidopsis thaliana*, and soya *Glycine max*, was studied. It was found that the stress–induced FDHs are at least 20 times more stable than the nonstress–induced PseFDH from *Pseudomonas * sp. 101 grown on methanol.

## INTRODUCTION

Formate dehydrogenase (EC 1.2.1.2, FDH), a NAD^+^–dependent enzyme, catalyses the oxidation of formate to carbon dioxide coupled to NAD^+ ^reduction into NADH.

NAD^+^ + HCOO^–^ → NADH + CO_2_^↑^

This is one of the key reactions providing the cell with NADH that is subsequently used for ATP synthesis. Formate dehydrogenases are very common in nature. They are present in various bacteria, such as methylotroph ic and symbiotic nitrogen–fixing bacteria [1]. In addition, FDH genes are found in many pathogenic microorganisms, including bacteria (*Staphylococcus aureus*, *Mycobacterium avium subsp. paratuberculosis str* . k10, various strains of *Bordetella* and *Legionella pneumophila* , *Francisella tularensis subsp. tularensis * SCHU S4) and microfungi ( *Histoplasma capsulatum * , * Cryptococcus neoformans var. neoformans* JEC21, etc.) [1]. Formate dehydrogenases are also present in various yeasts, nonpathogenic microfungi, mosses, and plants. These enzymes act as stress proteins in pathogenic microorganisms and plants, where the FDH content increases more than ten–fold under stress [2].

The FDH reaction mechanism is of great interest, as well as its physiological role. The enzyme belongs to the D–2–hydroxy–acid dehydrogenase superfamily [3]. The substrate structure, the formate ion, is the simplest among those of D–2–hydroxy–acid dehydrogenase substrates. This fact, along with the absence of an acid–base catalysis stage in the FDH enzyme cycle, makes FDH a model enzyme for the entire D–2–hydroxy–acid dehydrogenase superfamily.

FDH is extensively used in fine organic synthesis, being an ideal catalyst for cofactor regeneration. A number of processes to synthesise optically active compounds have been developed using NAD(P)H regeneration catalysed by FDH [4–7]; for example, Evonik (formerly Degussa) annually produces hundreds of tons of L–tert–leucine by this method [8]. Therefore, it is important to develop new FDH–based catalysts that can be stable not only in water solutions, but also in more aggressive media.

There are three main factors contributing to the overall FDH stability. The first one is the enzyme’s thermal stability; i.e. its ability to remain active at elevated temperatures. Thermal inactivation of enzymes usually occurs via protein denaturation. The thermal stability of formate dehydrogenases of different origins varies broadly. Thus, FDH from soybeans cloned in our laboratory [9], as well as FDH from bakery yeast, rapidly becomes inactive even at 45–46 °С. At the same time, FDH cloned from bacteria *Pseudomonas* sp.101 and * Staphylococcus aureus * , as well as from plants * Arabidopsis thaliana * , demonstrates good stability at 60–65 °С.

The second factor that determines the overall enzyme stability is its chemical stability; i.e. its ability to remain active in the presence of chemicals that modify protein amino acid residues, such as those located in the enzyme active site or responsible for the stabilization of the tertiary and quaternary structures. Cysteine residues are the most critical residues for formate dehydrogenases, since in almost all FDHs they play a significant role in the enzyme activity. In most cases, enzyme inactivation below 40 °С is caused either by chemical modification or oxidation of cysteine thiol groups.

The third factor particularly important for protein storage is the enzyme stability in the presence of proteases. Even the lowest levels of protease impurities (down to 0.001%) may cause a complete loss of enzyme activity during storage. No systematic studies on this issue have been conducted, although it has been shown that FDH from *Pseudomonas* sp.101 does not degrade under the action of *E. coli* proteases upon cultivation of the producer strain for 72 hours and longer.

Thermal stability of FDH has been well studied and described in the literature, even though information on the chemical stability of FDH remains rather scarce. The chemical–stability experiments have been performed on only a few enzymes, such as FDH from bacteria *Pseudomonas* sp.101 [10, 11] and * Mycobacterium vaccae * N10 [12] and yeast * Candida boidinii* [13]. The choice of a method to assess the enzyme chemical stability is very important. Several agents have been used to inactivate FDH, including Hg^2+^ and Cu^2+^ ions and specific reagents for cysteine thiol groups, such as 5,5’–Dithio–Bis(2–Nitrobenzoate) (DTNB) and p ‑ chloro mercury benzoate. However, no clear correlation between the chemical stability and the structure of the modifying agents has been established.

In this work, we chose hydrogen peroxide as an inactivation agent. This choice was dictated by the following reasons:

1. Hydrogen peroxide is a small molecule and can oxidise both surface thiols and those inside the protein globule. Bulky agents conventionally used for thiol modification, such as p ‑ chloro mercury benzoate and DTNB, for steric reasons, first rapidly react with the highly active Cys255, and then with other unidentified Cys residues, with a more than ten times slower rate [10].
2. Hydrogen peroxide is a *naturally occurring* inactivating and signalling agent. The natural origin and small size of hydrogen peroxide make it a good chemical agent for the assessment of FDH stability *in vivo*.
3. The concentration of hydrogen peroxide in the cell increases under stress conditions. Sometimes, the FDH concentration in the cell increases dramatically under stress, too. For instance, in plants, FDH is located in mitochondria, and under stress the enzyme content in the organelle can reach 9% of the total protein [14]. FDH from pathogenic * Staphylococcus aureus * is likewise a stress protein. In *S. aureus* biofilms, the concentration of FDH mRNA ranks third among all m–RNAs in the cell. Under these conditions, the level of FDH biosynthesis is 20 times higher than that in the bacteria growing as plankton [15]. These facts suggest that FDH induced under stress may be more stable in the presence of hydrogen peroxide than FDH synthesized under normal conditions; like FDH from *Pseudomonas* sp.101 grown on methanol.

The aim of this study was to compare the stability of wild–type FDH from various sources in the presence of hydrogen peroxide as an inactivating agent and to investigate the role of certain Cys residues in the chemical stability of FDH from methylotrophic bacterium *Pseudomonas * sp.101. The experimental plan consisted of the following tasks:

1. Study the kinetics of inactivation of a model enzyme in the presence of different concentrations of hydrogen peroxide and determine its kinetic mechanism;
2. Investigate the role played by certain Cys residues in the chemical stability of FDH from *Pseudomonas* sp.101; namely, Cys 145 in the formate–binding domain of the active site, Cys 255 in the coenzyme–binding domain located on the protein surface, and Cys 354 located outside the active site, on the protein surface;
3. Compare the chemical stability of FDH from bacteriun *Pseudomonas* sp.101 (enzyme synthesised in stress–free conditions) and *S. aureus* bacteria, plants *A. thaliana* and soya *Glicyne max* (enzymes overproduced under stress).

## EXPERIMENTAL

The preparations of recombinant formate dehydrogenase from bacterium *Pseudomonas* sp.101 (PseFDH) and its mutant forms with a single substitution Cys255Ala and double substitutions Cys145Ser/Cys255Ala and Cys255Ala/Cys354Ser, as well as recombinant wild–type FDH from bacterium *S. aureus* (SauFDH), plants *A. thaliana* (AraFDH) and soybeans * G. max * (SoyFDH), were kindly provided by Innovations and High Technologies MSU Ltd. (http://www.innotech–msu.com). All preparations were of 97–98% purity or higher as judged by sodium dodecyl sulfate–polyacrylamide gel electrophoresis.

*Determination of FDH activity*. FDH activity was determined spectrophotometrically by NADH absorption at 340 nm ( ε _340_ = 6220 М^–1^cm^–1^, Schimadzu UV 1601PC instrument, 30 °С, 0.1 М sodium phosphate buffer, pH 7.0). NAD^+^ and sodium formate were added at saturating concentrations of 1.5 mМ and 0.3 М, respectively.

*Inactivation of recombinant FDH by hydrogen peroxide*. Inactivation of FDH by hydrogen peroxide was performed in a 0.1 М sodium phosphate buffer, 0.01 М EDTA, at pH 7.0 and 25 °С. The hydrogen peroxide concentration was determined by absorption measurements at 240 nm ( ε _240_ = 43.6 М^–1^cm^–1^). For the experiments, 0.3 ml of H_2_O_2_ solution at varied fixed concentrations was added to 0.7 ml of FDH solution with activity of 2.5–4 units/ml. The temperature of the solutions was maintained at 25 ^о^С prior to mixing. The mixture was stirred vigorously and placed into a thermostat maintained at 25 ± 0.1 °С. At fixed time intervals, 20 µl samples were taken for residual activity measurements. Hydrogen peroxide working solutions were prepared by diluting a 33% H_2_O_2_ stock solution (9.1 mol) with bidistilled water.

## RESULTS AND DISCUSSION

**Inactivation of mutant PseFDH Cys255Ala by hydrogen peroxide at various concentrations**

The mutant enzyme from *Pseudomonas* sp.101 PseFDH GAV was used to study the dependence of the FDH inactivation rate on the hydrogen peroxide concentration. Two Cys residues at positions 145 and 255 are present in the PseFDH active site. Modification of either of these residues may lead to enzyme inactivation. It is possible that, by analogy with DTNB modification, hydrogen peroxide could demonstrate a different reactivity to the above–mentioned residues, since Cys145 is located inside the active site, whereas Cys255 is on the surface of the protein globule and exposed to the solution. We were mostly interested in studying the contribution of Cys145 into PseFDH inactivation by H_2_O_2_, therefore, a mutant PseFDH, with Cys255Ala substitution was used in these experiments.

**Fig. 1:**
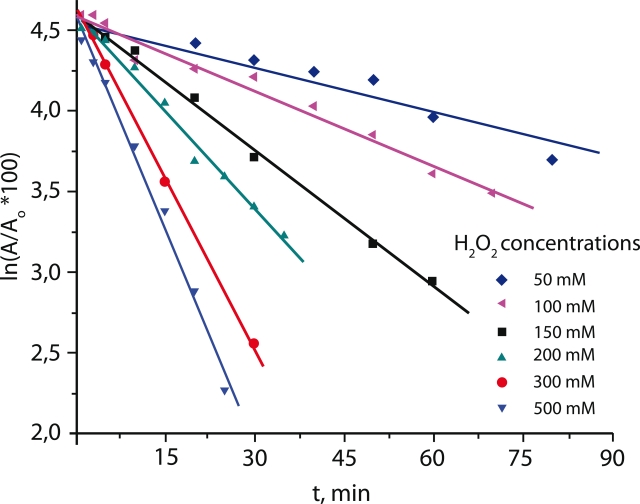
Inactivation of mutant PseFDH Cys255Ala at different hydrogen peroxide concentrations (0.1 М sodium phosphate buffer, pH 7.0, 25 °С)

We found an exponential decay dependence of the enzyme residual activity on time at all hydrogen peroxide concentrations used, as represented in linear semi–log graphs in Fig. 1. The effective kinetic constants of inactivation, *k^ef^_in_*, calculated from the slopes did not depend on the enzyme concentration.

Assuming a bimolecular mechanism for the reaction between the enzyme and H_2_O_2_,

FDH_act_ + Н_2_O_2_ → FDH_in_ + Н_2_O,

the reaction rate equation will look as follows:

*v_in_* = *k_in_* × [FDH_act_] × [H_2_O_2_],

where [FDH_act_] and [H_2_O_2_] are the active enzyme and hydrogen peroxide instant concentrations. Taking into account that [H_2_O_2_] >> [FDH_act_]_0_, the decrease in the H_2_O_2_ concentration in the course of the reaction is negligibly small; hence, [H_2_O_2_] ≈ [H_2_O_2_]_0_. In this case, the enzyme inactivation kinetics will be of the first order, with the effective first order constant

*k^ef^_in_* = *k_in_* × [H_2_O_2_]_0_.

**Fig. 2:**
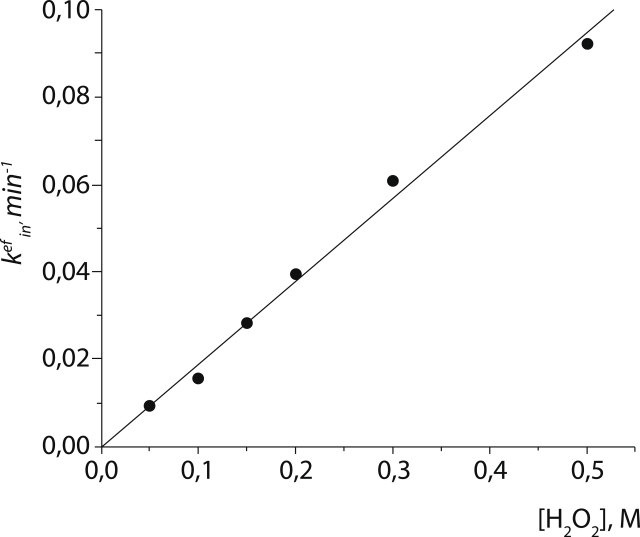
Dependence of the effective kinetic constant (k^ef^_in_) for mutant PseFDH Cys255Ala on the hydrogen peroxide concentration (0.1 М sodium phosphate buffer, pH 7.0, 25° С)

The true second–order constant of the inactivation kinetics can be derived from the dependence of the effective constant ( * k^ef^_in_* ) on the hydrogen peroxide concentration. Indeed, * k^ef^_in_* exhibits a linear dependence on the H_2_O_2_ concentration (Fig. 2). We obtained (3.17 ± 0.14)×10^–3^ М^–1^s^–1^ as the value of a bimolecular inactivation kinetics constant for PseFDH Cys255Ala.

Thus, our results show that the enzyme’s inactivation is a bimolecular reaction, and that the enzyme directly reacts with hydrogen peroxide with no intermediates formed. In the excess of hydrogen peroxide, the inactivation shows first–order kinetics; therefore, the effective first–order constant ( * k^ef^_in_* ) should not depend on the enzyme’s concentration (this has been confirmed experimentally). This allows us to use * k^ef^_in_* as a quantitative measure of FDH stability in experiments with FDH from different sources (or different mutant forms) at a constant H_2_O_2_ concentration. In all subsequent experiments, 0.15 М H_2_O_2_ was used.

## Inactivation of mutant PseFDHs by hydrogen peroxide

**Fig. 3:**
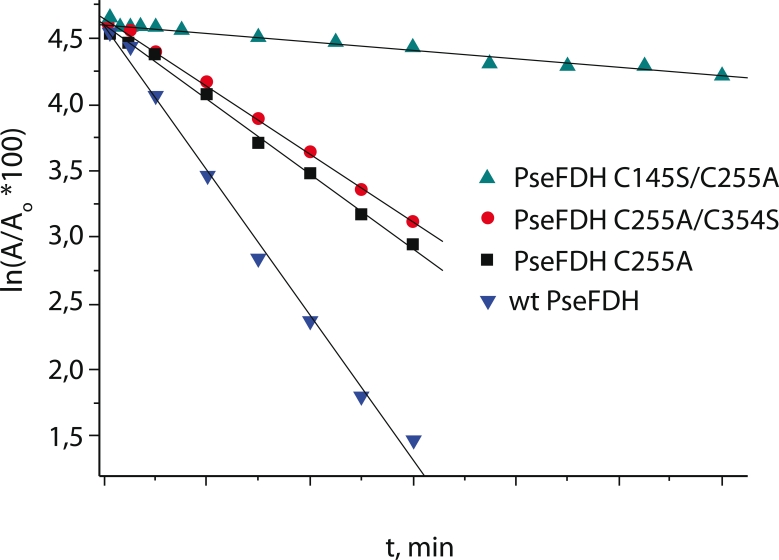
Inactivation by hydrogen peroxide of the wild-type PseFDH (wt-PseFDH) and its various mutant forms (0.15 М H_2_O_2_, 0.1 М sodium phosphate buffer, pH 7.0, 25 °С)

The results of hydrogen peroxide inactivation of the wild–type and three mutant FDH enzymes from *Pseudomonas* sp.101 with Cys substituted at various positions are presented in Fig. 3. We had previously prepared a number of mutant PseFDH enzymes with various substitutions for Cys 145, 255, and 354. For this study, we selected only those mutants that had demonstrated the best kinetic behavior.

The hydrogen peroxide inactivation of all FDH forms studied showed first–order kinetics. As we expected, the wild–type enzyme was the least stable (Fig. 3). The substitution of Cys255 with Ala reduces the inactivation constant by a factor of two ( * k^ef^_in_* = 9.13×10^–4^ and 4.69×10^–4^ s^–1^, respectively). In the other mutant PseFDH, the Cys354Ser substitution was added to Cys255Ala. The substitution of Cys354 with Ser had only a negligible effect on the enzyme stability towards H_2_O_2_, a confirmation of our conclusion that the Cys354 residue played no significant role in the enzyme activity [1]. The highest stability was demonstrated by the mutant PseFDH with substitutions in the enzyme active site, Cys145Ser and Cys255Ala.

The comparison of the effective inactivation kinetic constants ( *k^ef^_in_* ) for the wild–type PseFDH and mutant Cys255Ala and Cys145Ser/Cys255Ala PseFDHs (Table 1) provides an estimate for the contribution of each of these residues into the enzyme stability. The contribution of the Cys255 residue to the value of * k^ef^_in_* is 4.40×10^–5^ s^–1^ calculated as the difference between the * k^ef^_in_* values for the wild–type and Cys255Ala PseFDH. The contribution of the Cys145 residue to the value of *k^ef^_in_* is 4.17×10^–5^ s^–1^ calculated as the difference between the * k^ef^_in_* values for Cys255Ala and Cys145Ser/Cys255Ala PseFDHs. These calculations indicate the similar reactivity of these two residues towards hydrogen peroxide, despite their different locations in the protein. As mentioned above, the difference between the reactivities of the Cys145 and Cys255 residues was at least 2–3 orders of magnitude when DTNB was used as a modifying agent [10].

**Table 1:** Effective first-order kinetic constants for hydrogen peroxide inactivation of the wild-type and mutant PseFDHs (0.15 М hydrogen peroxide, 0.1 М sodium phosphate buffer, pH 7.0).

Enzyme	Wild-type PseFDH	PseFDH C255A	PseFDH C255A/C354S	PseFDH C145S/C255A
k^ef^_in_×10^-5^ (s^-1^)	91.3 ± 3.2	46.9 ± 1.2	42.5 ± 0.8	5.20 ± 0.37

## Inactivation of FDH from bacteria and plants by hydrogen peroxide

**Fig. 4:**
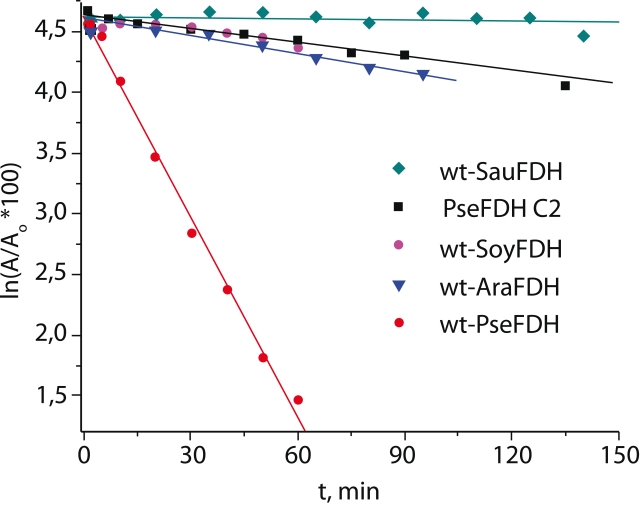
Inactivation by hydrogen peroxide of formate dehydrogenases from different sources. wt-SauFDH, wt-PseFDH, wt-SoyFDH, and wt- AraFDH are wild-type recombinant FDHs from bacteria S.aureus and Pseudomonas sp.101, soya Glycine max and plant A.thaliana, respec- tively. PseFDH C2 is mutant PseFDH Cys145Ser/Cys255Ala (0.15 М H_2_O_2_, 0.1 М phosphate buffer, pH 7.0, 25° С).

We studied the stability of FDH from various sources to inactivation by hydrogen peroxide. We selected three FDH enzymes, the biosynthesis of which increases sharply under stress conditions: SauFDH of bacterial origin and AraFDH and SoyFDH of higher plants origin (Fig. 4). The wild–type FDH from *Pseudomonas* sp.101 and its most stable to hydrogen peroxide inactivation Cys145Ser/Cys255Ala PseFDH mutant were used for reference. PseFDH is not a stress protein; its biosynthesis is induced in bacterium *Pseudomonas* sp.101 under grown on methanol. The stress–induced FDH of plant, as well as bacterial origin, shows very high stability to inactivation induced by hydrogen peroxide (Fig. 4). They are much more stable than the wild–type PseFDH; only the best mutant, PseFDH Cys145Ser/Cys255Ala, has a stability comparable to those of FDH of plant origin. Although plant FDHs show almost identical stability to inactivation by hydrogen peroxide, they differ in their thermal stability by more than 5,000 times [9]. FDH from pathogenic bacterium *S. aureus* was the most stable to inactivation by hydrogen peroxide. As shown in Fig. 4, the residual activity of this enzyme after 4 hours of incubation in the presence of 0.15М H_2_O_2_ was more than 90%. SauFDH also possesses high thermal stability, being second only to PseFDH among all known formate dehydrogenases.

The results obtained in inactivation experiments provide evidence for the activity loss upon treatment with hydrogen peroxide for all the enzymes studied, even those without Cys residues in the active sites, which inactivate more slowly (Fig. 4). The values of the effective inactivation kinetic constants for single mutant PseFDH Cys255Ala and double mutant Cys255Ala/Cys354Ser (Table 1) suggest that, most likely, their activity decreases not due to the oxidation of the Cys residues located outside the enzyme active site, but rather because of the modification of other amino acid residues in the active site. This means that hydrogen peroxide is not a reagent specific for Cys residues in FDH. It is this nonspecificity of H_2_O_2 _that reveals the contribution of other amino acid residues into FDH stability; these residues upon oxidation result in a decreased enzymatic activity. Using the *k^ef^_in_* value, one can quantify the contribution of those residues into the enzyme chemical stability. The contribution of non–cysteine residues is 6 times lower in the case of FDH of plant origin than in the case of SauFDH (Fig. 4).

The higher stability of SauFDH to hydrogen peroxide–induced inactivation compared to that of FDH of plant origin correlates well with the stability requirements for these enzymes under stress *in vivo* . Plant cells can withstand much milder stress conditions for a much shorter period of time before dying than the conditions in which the *S. aureus * biofilms can survive. Apparently, the better stress resistance of *S. aureus* must be supported by the high stability of all cell components responsible for survival under stress, including FDH. Therefore, our results confirm the hypothesis put forward in the introduction; that FDHs upregulated under stress conditions should be more stable with respect to inactivation by hydrogen peroxide: the stronger the stress, the higher the stability. In conclusion, the experiments on the inactivation of purified FDH preparations by hydrogen peroxide can be used for comparative analysis of the stability of formate dehydrogenases *in vivo*.
